# Neural Excitability and Singular Bifurcations

**DOI:** 10.1186/s13408-015-0029-2

**Published:** 2015-08-06

**Authors:** Peter De Maesschalck, Martin Wechselberger

**Affiliations:** Hasselt University, Agoralaan gebouw D, 3590 Diepenbeek, Belgium; School of Mathematics & Statistics, University of Sydney, F07, NSW 2006 Sydney, Australia

**Keywords:** Bifurcation theory, Canards, Excitability, Geometric singular perturbation theory, Neural dynamics, 34E15, 34E17, 34K18, 37N25, 92B25

## Abstract

We discuss the notion of excitability in 2D slow/fast neural models from a geometric singular perturbation theory point of view. We focus on the inherent singular nature of slow/fast neural models and define excitability via *singular bifurcations*. In particular, we show that type I excitability is associated with a novel *singular Bogdanov–Takens/SNIC* bifurcation while type II excitability is associated with a singular Andronov–Hopf bifurcation. In both cases, canards play an important role in the understanding of the unfolding of these singular bifurcation structures. We also explain the transition between the two excitability types and highlight all bifurcations involved, thus providing a complete analysis of excitability based on geometric singular perturbation theory.

## Excitable Systems

Most neurons are excitable, i.e. they are typically silent but can fire an action potential or produce a firing pattern in response to certain forms of stimulation. The fact that equivalent stimulation can elicit qualitative different spiking patterns in different neurons demonstrates that intrinsic coding properties differ significantly from one neuron to the next.

**Fig. 1:**
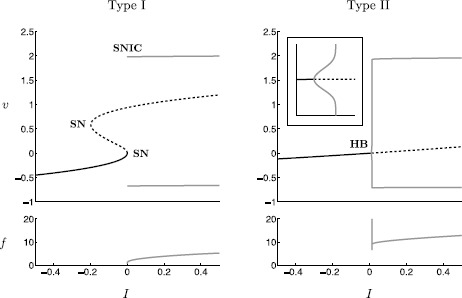
Bifurcation diagrams of the canonical model (1) together with ‘frequency–current’ (f–I) plots: (*Type I*) $c=0.005$; SNIC bifurcation near $I=I_{\mathrm{bif}}=0$ where the frequency approaches zero; (*Type II*) $c=4$; supercritical singular Andronov–Hopf bifurcation near $I=I_{\mathrm{bif}}=0$; the subsequent canard explosion is clearly visible; note the small frequency band for the relaxation oscillation branch

A first answer to the question of the neuron’s computational properties was given by Hodgkin [1] in the 1940s, who identified three basic types (classes) of excitable axons distinguished by their different responses to injected steps of currents of various amplitudes. *Type I (class I)* axons are able to *integrate* the input strength of an injected current step, i.e. the corresponding *frequency–current (f–I) curve* is continuous (see Fig. 1). *Type II (class II)* axons have a discontinuous f–I curve because of their inability to maintain spiking below a certain frequency. The frequency band of a type II neuron is very limited and, hence the frequency is relatively insensitive to the strength of the injected current. It appears that type II neurons *resonate* with a preferred frequency input. *Type III (class III)* axons will only fire a single or a few action potentials at the onset of the injected current step, but are not able to fire repetitive action potentials like type I and type II neurons. Type III neurons are able to *differentiate*, i.e. they are able to encode the occurrence of a ‘change’ in the stimulus. Such *phasic* firing (versus *tonic* or repetitive firing) identifies these type III neurons as *slope detectors* [2]. Obviously, the f–I curve is not defined for type III neurons.

Starting in the 1980s, Rinzel and Ermentrout [3, 4] pioneered a mathematical framework based on bifurcation theory that distinguishes type I and type II neural models. Recall, type I and type II neurons are able to fire trains of action potentials (tonic firing) if depolarised sufficiently strong which distinguishes them from type III neurons. This distinction points to a bifurcation in type I and type II neurons where the cell changes from an excitable to an oscillatory state. The main bifurcation parameter is given by *I*, the magnitude of the current step protocol, and leads to the following classical definition of excitability via bifurcation analysis under the variation of the applied current *I* [4]:

- Type I: The stable equilibrium (resting state) disappears via a *saddle-node on invariant circle (SNIC)* bifurcation.
- Type II: The stable equilibrium (resting state) loses stability via an *Andronov–Hopf bifurcation (HB)*, either sub- or supercritical.

Note that there is no bifurcation occurring in type III models, i.e. the equilibrium (resting state) remains stable. For a possible biophysical interpretation of these three distinct excitability types we refer to, e.g., [5, 6].

A canonical model that captures the main features of neural excitability is given by

1 $$ \begin{aligned} w' &= \varepsilon {g}(w,v)=\varepsilon \bigl(G(v)-w\bigr), \\ v' &= f(w,v,I)= v^{2}(d-v)-w+I , \end{aligned} $$

with

2 $$ G(v) = \textstyle\begin{cases} cv, & v\le v_{\mathrm{th}},\\ cv +e(v-v_{\mathrm{th}})^{2}, & v>v_{\mathrm{th}}, \end{cases} $$

a sufficiently smooth (here $C^{1}$) function with threshold parameter $v_{\mathrm{th}}$, and parameters $0<\varepsilon \ll1$, $d,e>0$ such that $\frac {d}{3} < e < d$ holds^1^ and $c \in[0,c_{1}]$. The parameter $I\in[I_{0},I_{1}]\subset \mathbf{R}$ is the main bifurcation parameter and is often associated with the injected current in a real neuron. In this canonical model, we are able to identify all three excitability types. Figure 1 shows bifurcation diagrams and frequency–current plots for type I and type II excitability where the parameters $v_{\mathrm{th}}=0.15$, $\varepsilon =10^{-2}$, $d=2$, $e=1.5$ are fixed and $(I,c)$ are varied.

### Remark 1

In the canonical model (1), the nonlinear nature of $G(v)$ is essential to guarantee relaxation type behaviour as observed in type I neurons which distinguishes this polynomial model from the classic FitzHugh–Nagumo model [7, 8] which can only produce type II (and type III) behaviour. In more biophysically inspired two-dimensional systems, the Morris–Lecar model [9] presents a prime example which is able to produce all three excitability types; see also [4–6, 10].

### Slow–Fast Excitable Systems

An important feature of most neural systems is that they evolve on *multiple time scales*; see, e.g., [11]. It is the interplay of the dynamics on different temporal scales that creates complicated rhythms. Multiple (or slow–fast) time-scale problems are usually modelled by *singularly perturbed systems* such as (1) where the time-scale separation of the ‘fast’ variable *v* (voltage) and the ‘slow’ variable *w* (recovery variable) is explicitly identified through the singular perturbation parameter $\varepsilon \ll1$. The interest in such slow–fast systems goes towards the presence of so-called *relaxation oscillations*. Along an orbit of relaxation oscillation type, parts where the velocity of the phase variables is small (the slow parts) are alternated with high velocity peaks on short time intervals (the fast parts). During the slow parts, the phase state is $O(\varepsilon )$-close to the *critical set*$f(w,v,I)=0$ (because then $\Vert (w',v')\Vert = O(\varepsilon )$), whereas during the fast part the phase state is at an $O(1)$-distance from this critical set. Tonic firing as observed in (1) is exactly of relaxation type.

We want to emphasise this inherent slow–fast time-scale structure found in many neuronal models and use geometric singular perturbation theory (GSPT) [12, 13] as a mathematical framework. In this approach, we focus on a class of two-dimensional singularly perturbed models given by

3 $$ \begin{aligned} w' &= \varepsilon g(w,v,\varepsilon , \lambda), \\ v' &= f(w,v,\varepsilon ,I), \end{aligned} $$

where $I\in[I_{0},I_{1}]\subset \mathbf{R}$ is an external (constant) drive of the excitable system, the prime denotes the (fast) time derivative $d/dt$ and $\varepsilon \ll1$ is a small positive parameter encoding the time-scale separation between the slow and fast variables. The parameter *λ* is considered in some interval $[\lambda_{0},\lambda_{1}]$ and will serve, together with *I*, as a bifurcation parameter. The functions *f* and *g* are assumed to be sufficiently smooth. We stress that it is not important for *f* to be independent of *λ* and for *g* to be independent of *I*, though it does simplify the presentation.^2^

By switching from the fast time scale *t* to the slow time scale $\tau = \varepsilon t$, system (3) transforms to

4 $$ \begin{aligned} \dot{w} &= g(w, v, \varepsilon ,\lambda), \\ \varepsilon \dot{v} &= f(w, v,\varepsilon ,I) , \end{aligned} $$

where the overdot denotes the (slow) time derivative $d/d\tau$. As $\varepsilon \to0$, the trajectories of (3) converge during fast segments to solutions of the one-dimensional *layer (or fast) problem*

5 $$ \begin{aligned} w^{\prime}&= 0, \\ v^{\prime}&= f(w, v, 0,I), \end{aligned} $$

while during slow segments, trajectories of (4) converge to solutions of

6 $$ \begin{aligned} \dot{w} &= g(w, v, 0), \\ 0 &= f(w,v, 0,I), \end{aligned} $$

which is a one-dimensional differential-algebraic problem called the *reduced (or slow) problem*. Note that the critical set

7 $$ S := \bigl\{ (w, v) \in \mathbf{R}\times \mathbf{R}\vert f(w, v, 0,I) = 0 \bigr\} $$

is the set of equilibria of (5). In general, this set *S* defines a differentiable manifold referred to as the *critical manifold*, and it forms the phase space of the reduced problem (6). GSPT [12–15] uses these lower one-dimensional sub-systems (5) and (6) to predict the dynamics of the full two-dimensional system (3) or (4) for $\varepsilon > 0$. In Sect. 2, we provide the general setup for a class of two-dimensional systems in the context of GSPT that covers all three excitability types.

While this GSPT approach to explain relaxation type behaviour in neural systems is well known to the mathematical and computational neuroscience community, see e.g. [5, 11], it is not often or consequently used to explain the underlying bifurcation structure in such singularly perturbed systems. This is the focus of Sects. 3–6 and we study *singular bifurcations* and their unfoldings in a normal form introduced in Sect. 3.1.

A closer look at the bifurcation diagram of type II excitability in Fig. 1 reveals that shortly after the Andronov–Hopf bifurcation the amplitude of the bifurcating limit cycles explodes dramatically under a very tiny (an exponentially small) parameter change. This is known as a *canard explosion* [14, 15] and indicates that the singular perturbation nature of the neural model is also reflected in the bifurcation structure. Note also a similar dramatic change in frequency near this *singular* Andronov–Hopf bifurcation. We will review this (well-known) bifurcation phenomenon in Sect. 4.

Additional bifurcation structure is necessary to explain the transition from type II to type I excitability. This is covered in Sect. 5 where an *incomplete* canard explosion is identified. This lesser-known phenomenon refers to a premature termination of a canard explosion in a homoclinic bifurcation.

Similarly to the singular Andronov–Hopf bifurcation, one has to expect that the SNIC bifurcation associated with type I excitability shown in Fig. 1 must have a singular nature. We identify a *singular Bogdanov–Takens/SNIC* bifurcation point as the organising centre for type I excitability. Unfolding this type I singular bifurcation structure is the main focus of Sect. 6 which is based on the blow-up method, a desingularisation technique for nilpotent singularities that has been successfully implemented in geometric singular perturbation problems with loss of normal hyperbolicity [14–16].

Finally, we summarise our results in Sect. 7 and discuss its implications for possible numerical observations in slow–fast neural models.

## The Setup for Slow–Fast Excitable Systems

We start with introducing basic assumptions on the singularly perturbed system (3), respectively, (4).

### Assumption 1

**Fig. 2:**
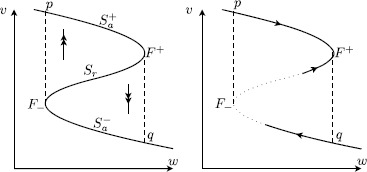
*Left*: several branches of the critical manifold, with fold points $F^{\pm}$ in between. *Right*: slow dynamics on the cubic. The nullcline $\dot{w}=0$ may intersect the cubic one or more times along the dotted part of this cubic

For each $I\in[I_{0},I_{1}]$, the critical manifold *S* is cubic shaped and given as a graph $\{w=\varphi _{I}(v)\}$, i.e.

$$S=S_{a}^{-} \cup F^{-} \cup S_{r} \cup F^{+} \cup S_{a}^{+} , $$

with attracting outer branches $S_{a}^{\pm}$, repelling middle branch $S_{r}$, and folds $F^{\pm}$. We also assume that the vertical fibres containing the two local folds $F^{-}$, respectively, $F^{+}$ intersect the critical manifold one more time outside the fold points at points *p*, respectively, *q*; see Fig. 2. The two folds are assumed to be regular extremes of the graph $w=\varphi _{I}(v)$.

The representation of *S* as a graph $\{w=\varphi _{I}(v)\}$ necessarily implies that $\partial f/\partial v \neq0$ for all $(w,v)\in S$. The stability condition on the branches $S_{a}^{\pm}$ and $S_{r}$ identifies them as normally hyperbolic; see Fig. 2 (left). This refers to the stability property of *S* as a set of equilibria of the layer problem (5) and is expressed by

$$\textstyle\begin{cases} \frac{\partial f}{\partial v}(w,v,0,I) < 0 & \mbox{for }(w,v)\in S_{a}^{\pm}, \\ \frac{\partial f}{\partial v}(w,v,0,I) > 0 & \mbox{for }(w,v)\in S_{r}. \end{cases} $$

The two local extremes of *S* denoted by $(w^{\pm},v^{\pm})=(\varphi _{I}(v^{\pm}),v^{\pm})$ are called fold points $F^{\pm}$; they correspond to saddle-node bifurcation points in the layer problem (5) and we have

8 $$\begin{aligned} \frac{\partial f}{\partial v}\bigl(w^{+},v^{+},0,I\bigr) &= 0, \qquad \frac{\partial^{2} f}{\partial v^{2}} \bigl(w^{+},v^{+},0,I\bigr) < 0, \end{aligned}$$

9 $$\begin{aligned} \frac{\partial f}{\partial v}\bigl(w^{-},v^{-},0,I\bigr) &= 0, \qquad \frac{\partial^{2} f}{\partial v^{2}} \bigl(w^{-},v^{-},0,I\bigr) > 0. \end{aligned}$$

Figure 2 can be viewed as the corresponding bifurcation diagram of the layer problem (5) with *w* as the main bifurcation parameter. If we want to impose the geometry shown in Fig. 2, then we need to—and will—assume that

10 $$ \frac{\partial f}{\partial w}< 0 ,\quad(w,v)\in S. $$

### Assumption 2

For each $\lambda\in[\lambda_{0},\lambda_{1}]$, the following holds along the *w*-nullcline $g(w,v,0,\lambda)=0$ of system (4):

$$\frac{\partial g}{\partial w}\neq0 ,\qquad \frac{\partial g}{\partial v}\cdot\frac{\partial g}{\partial w} \le0 . $$

This assumption implies that the *w*-nullcline is a graph $\{w=\psi _{\lambda}(v)\}$, and $\psi_{\lambda}$ is a monotonically increasing function. While mathematically not necessary, this reflects the property of a typical neural model where $g=0$ is given as a graph of a sigmoidal function over *v*.

### Assumption 3

**Fig. 3:**
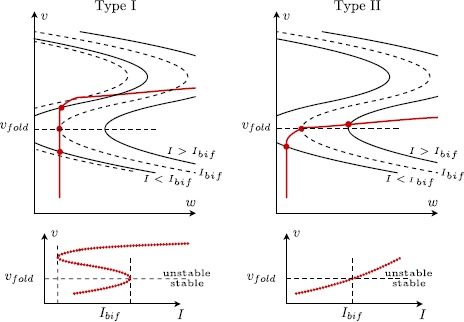
Neural model (1): nullclines under variation of *I* which leads to the (singular limit) definition of $I_{\mathrm{bif}}$: (*Type I*) $I=I_{\mathrm{bif}}$ at a saddle-node bifurcation; (*Type II*) $I=I_{\mathrm{bif}}$ at (singular) Andronov–Hopf bifurcation. In the transition from type II to type I, a cusp bifurcation appears as can be predicted by looking from *the bottom pictures in this figure*

For all $(I,\lambda)\in[I_{0},I_{1}]\times[\lambda_{0},\lambda_{1}]$, system (4) can have one, two or three equilibria on $w=\varphi _{I}(v)$, all of them located either on $S_{r}$ or on $S_{a}^{-}$; see Fig. 3. The number of equilibria and their exact locations depend on $(I,\lambda)$.

This assumption together with the geometric properties of the two nullclines $f=0$ and $g=0$ defined in Assumptions 1 and 2 imply the following under the variation of $(I,\lambda)\in[I_{0},I_{1}]\times[\lambda_{0},\lambda_{1}]$; see Fig. 3:

- there can be no more than one equilibrium on the lower attracting branch $S_{a}^{-}$;
- the existence of exactly two equilibria indicates a saddle-node bifurcation either on $S_{r}$ or at $F^{-}$.

### Remark 2

Assumption 3 sets the scene for the transition from an excitable to an oscillatory state. It excludes the possibility of a transition from an oscillatory state to a(nother) steady state (known as depolarisation block in neuroscience), by restricting the parameter space from above. This is not necessary but allows us to focus on the onset of oscillations, not the termination.

Next we look at the reduced problem (6). The corresponding one-dimensional dynamics on the critical manifold *S* projected onto its base coordinate *v* is given by

11 $$ -\frac{\partial f}{\partial v}\bigl(\varphi _{I}(v),v,0,I\bigr) \dot{v}=\frac {\partial f}{\partial w}\bigl(\varphi _{I}(v),v,0,I\bigr) g\bigl( \varphi _{I}(v),v,0,\lambda\bigr) . $$

Generically, system (11) defines the reduced dynamics along the hyperbolic branches of the critical manifold, outside the fold points $F^{\pm}$ where (11) is singular. Assumption 3 implies that $g(\varphi _{I}(v),v,0,\lambda)\neq0$ on the upper branch $S_{a}^{+}$.

### Assumption 4

For all $(I,\lambda)\in[I_{0},I_{1}]\times[\lambda_{0},\lambda_{1}]$, the fold point $F^{+}=(w^{+},v^{+})$ is a regular jump point. More precisely, we impose

12 $$ \frac{\partial^{2} f}{\partial v^{2}}\bigl(w^{+},v^{+},0,I\bigr)\cdot \frac{\partial f}{\partial w} \bigl(w^{+},v^{+},0,I\bigr) >0, \qquad g\bigl(w^{+},v^{+},0,\lambda\bigr) > 0. $$

The first condition in (12) is equivalent to imposing the requirement that $\varphi _{I}'(v)=0$, $\varphi _{I}''(v)<0$; in other words, the fold $F^{+}$ is a regular local maximum of $\varphi _{I}$.^3^ Together with the second condition, this determines the direction of the reduced flow near $F^{+}$ and qualifies this fold point as a jump point: all orbits that come in along the upper branch $S_{a}^{+}$ jump off the fold $F^{+}$ and follow the fast fibre towards *q*; see Fig. 2 (right). Note that no equilibrium on $S_{r}$ can approach the upper fold $F^{+}$ while varying parameters (see also Remark 2).

**Fig. 4:**
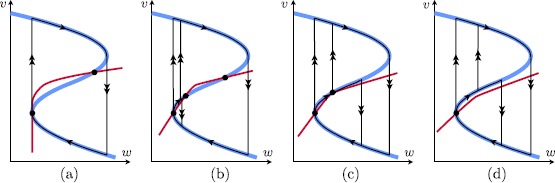
Singular limit bifurcations at the lower fold $F^{-}$ and their singular limit orbits in system (1) ($v_{\mathrm {th}}>0$, $I=I_{\mathrm{bif}}=0$). (**a**) (*Type I*) singular saddle-node homoclinic (SNIC) ($c=0$) together with a singular Bogdanov–Takens bifurcation (= singular BT/SNIC); (**b**) singular Andronov–Hopf bifurcation with incomplete family of canard cycles ($0< c< c_{\mathrm {sn}}$); (**c**) family of (incomplete) canard cycles and family of singular saddle-node homoclinics of canard type ($c=c_{\mathrm{sn}}$); (**d**) (*Type II*) singular Andronov–Hopf bifurcation and (complete) family of canard cycles ($c>c_{\mathrm{sn}}$)

On the other hand, an equilibrium from $S_{a}^{-}$ may cross or bifurcate at the lower fold $F^{-}$, which is necessary to observe a bifurcation from an excitable to an oscillatory state. Assumptions 3 and 4 imply that the reduced flow on $S_{a}^{-}$ is either towards an equilibrium on $S_{a}^{-}$ or towards the lower fold $F^{-}$ (see Fig. 2 (right) and Fig. 4), another essential feature for an excitable/oscillatory system.

These basic assumptions hold for many two-dimensional neuronal models including the canonical system (1). This model has a cubic-shaped critical manifold *S* (Assumption 1). The function $G(v)$ is monotonically increasing (Assumption 2). It is the nonlinear nature of *G* imposed by Assumption 3 that allows us to explore the cases of different numbers of equilibria (restricted to $S_{a}^{-}$ and $S_{r}$ only). If $0< I_{1}< I_{F^{+}}$ and $v_{\mathrm{th}}< v^{+}$, where $I_{F^{+}}$ is defined implicitly by $\varphi _{I_{F^{+}}}(v^{+})=G(v^{+})$, then Assumptions 3 and 4 are fulfilled and $F^{+}$ is a regular jump point for all $I\in[I_{0},I_{1}]$ and $c \in[0,c_{1}]$.

## Singular (or Slow–Fast) Bifurcations

Our main task is to identify bifurcation structures in the singularly perturbed system (4) based on *singular* observations, i.e. for $\varepsilon =0$. For system (1) we have $F^{-}=(\varphi _{I}(v^{-}),v^{-})=(I,0)$. At this fold, $G(v)-w = G(0)-I$ is zero when $I=I_{\mathrm{bif}}=G(0)$:

13 $$ I_{\mathrm{bif}} = \textstyle\begin{cases} 0, & v_{\mathrm{th}} \geq0, \\ ev_{\mathrm{th}}^{2}, & v_{\mathrm{th}} < 0. \end{cases} $$

When $I< I_{\mathrm{bif}}$, there is a unique singularity on the stable branch $S_{a}^{-}$ with possible extra singularities on the middle branch while for $I>I_{\mathrm{bif}}$ there is no singularity on $S_{a}^{-}$. Hence, the transition from an excitable to an oscillatory state happens near $I\sim I_{\mathrm{bif}}$ and we will distinguish different excitability types by different behaviour near the lower fold $F^{-}$.

### Remark 3

System (1) models type III excitability if $I_{\mathrm {bif}}$ lies outside of the interval $[I_{0},I_{1}]$, i.e. $I_{\mathrm {bif}}>I_{1}$. In general, type III excitability can be characterised by the property that $I_{\mathrm{bif}}$ lies outside of the interval $[I_{0},I_{1}]$.

### Normal Form Near the Singular Fold Point

In the canonical model (1) assuming it is not of type III, the fold $F^{-}$ is a singular fold point at $I=I_{\mathrm{bif}}$ defined in (13). We can make the simple coordinate transformations $w=x/d+I$ and $v=y/d$ to write the system in the form

$$\begin{aligned} x' &= \varepsilon \bigl(dG(y/d)-x-dI\bigr), \\ y' &= y^{2}-x-y^{3}/d^{2} , \end{aligned} $$

which simplifies for $v\leq v_{\mathrm{th}}$ to

14 $$ \begin{aligned} x' &= \varepsilon (cy-x-a), \\ y' &= y^{2}-x-y^{3}/d^{2} , \end{aligned} $$

where $a=dI$. Note that, when $v>v_{\mathrm{th}}$, an extra term $\varepsilon \frac {e}{d}(y-dv_{\mathrm{th}})^{2}$ appears in the $x'$ equation.

While in general one has to do a bit more work, the normal form for system (3) near the singular fold $F^{-}$ shows similarity with the normal form (14) of the canonical model. The local shape of the vector field (3) near the fold $F^{-}$ is described in the following proposition.

#### Proposition 1

*Under Assumptions*1*–*3*and* (10), *the family of vector fields* (3) *can be locally transformed in the following normal form near*$F^{-}$:

15 $$ \begin{aligned} x' &= \varepsilon \bigl(cy - \sigma x -a + O\bigl(x^{2},y^{3},xy,\varepsilon y^{2}\bigr) \bigr), \\ y' &= y^{2}-x + \beta y^{3} + O \bigl(y^{4}\bigr) , \end{aligned} $$

*where*$\sigma=\pm1$, $\sigma c\geq0$*and*$\beta\neq0$. *The coefficientsa*, *candβcan be computed explicitly in terms of*$(I,\lambda ,\varepsilon )$.

#### Proof

Starting with system (3), and working under Assumption 1 and (10), we know from the implicit function theorem that the *v*-nullcline $f(w,v,0,I)=0$ is a graph $w=\varphi _{I}(v)$. By the same argument, we can factor $f(w,v,\varepsilon ,I)$ as $(\tilde{\varphi }_{I,\varepsilon }(v)-w)\cdot\tilde{f}$ for some strictly positive function $\tilde{f}(w,v,\varepsilon ,I)$, and with $\tilde{\varphi }_{I,\varepsilon }(v) = \varphi _{I}(v) + O(\varepsilon )$. By dividing the vector field by $\tilde{f}$ (i.e. by rescaling time), we obtain the (topologically) equivalent vector field^4^

$$\begin{aligned} w' &= \varepsilon \tilde{g}(w,v,\varepsilon ,\lambda,I), \\ v' &= \tilde{\varphi }_{I,\varepsilon }(v)-w . \end{aligned} $$

The reader may verify that Assumptions 1–3 remain unchanged after this manipulation.^5^ Since $\varphi _{I}''(v)>0$ and hence $\tilde{\varphi }_{I,\varepsilon }''(v)>0$, we can solve $\tilde{\varphi }_{I,\varepsilon }'(v)=0$ using the implicit function theorem to find an *I*-dependent point at $v=v_{*}(I,\varepsilon )$, *ε*-close to the fold of $\varphi _{I}(v)$.^6^ Replacing *v* by $v-v_{*}$ and *w* by $w-\tilde{\varphi }_{I,\varepsilon }(v_{*})$ allows one to assume in the remainder that the fold of $\tilde{\varphi }_{I,\varepsilon }(v)$ is located at the origin. It suffices to see that after this translation, $v' = B_{0}(\varepsilon )v^{2} - w + O(v^{3})$ for some $B_{0}(\varepsilon )>0$. We arrive at

$$\begin{aligned} w' &= \varepsilon \bigl(A_{0} + A_{1}v + A_{2}v^{2} + A_{3}w + O\bigl( \varepsilon ,w^{2},v^{3},wv\bigr) \bigr), \\ v' &= B_{0}(\varepsilon )v^{2}-w + O \bigl(v^{3}\bigr), \end{aligned} $$

where the coefficients $A_{k}$ and $B_{0}$ depend on $(I,\lambda)$. Let us now write $(w,v) = (Zx + \varepsilon Py + \varepsilon ^{2}Q,v=Zy + \varepsilon R)$ for well-chosen $(P,Q,Z,R)$. Aided by a computer algebra program it is easy to verify that one can choose $(P,Q,Z)$ in terms of *R* to make sure $y' = y^{2}-x +O(y^{3})$. Once we have obtained this, it is not so hard to see that one can choose *R* in such a way that we arrive at

$$\begin{aligned} x' &= \varepsilon \bigl(C_{0} + C_{1}y + C_{2}x + O\bigl(\varepsilon ,x^{2},y^{3},xy \bigr) \bigr), \\ y' &= y^{2}-x + B_{1}y^{3}+ O \bigl(y^{4}\bigr) . \end{aligned} $$

In other words, the leading-order coefficient with $y^{2}$ in $x'$ has disappeared. The $O(\varepsilon )$ terms in $x'$ that are not part of $O(x^{2},y^{3},xy)$ are of the form $\nu_{1}(\varepsilon )$, $\nu_{1}(\varepsilon )x$, $\nu _{2}(\varepsilon )y$, $\nu_{3}(\varepsilon )y^{2}$. The first three terms can be put together with the primary part $C_{0}+C_{1}y+C_{2}x$, by allowing $C_{i}$ to be *ε*-dependent. It shows that the remainder term is $O(x^{2},y^{3},xy,\varepsilon y^{2})$. A linear rescaling of $(x,y,t)$ allows one to further reduce to the case $C_{2}=\pm1$. The constraint $C_{0}C_{2}\ge0$ follows from Assumptions 2–3. □

#### Remark 4

For system (14), the coefficients in the normal form are given by $c\ge0$ and

16 $$ a=dI,\qquad\beta=-1/d^{2},\qquad\sigma=1, $$

at least when $v_{\mathrm{th}}>0$. When $v_{\mathrm{th}}<0$, the parameters *a* and *c* are shifted by extra terms (which we will not give details of here) that depend on $v_{\mathrm{th}}$. The extra term in the coefficient *a* will make $a=0$ at $I=I_{\mathrm{bif}} = ev_{\mathrm {th}}^{2}$. The coefficient *c* in the normal form in that case depends regularly on $v_{\mathrm{th}}$ and can be used to locally distinguish the different scenarios depicted in Fig. 3. Hence, it makes sense to consider the coefficients $(a,c)$ in the normal form as the main bifurcation parameters.

In system (15) for $a<0$, there exists a stable equilibrium on the lower stable branch $S_{a}^{-}$ while for $a>0$, there is no stable equilibrium on the lower stable branch $S_{a}^{-}$ and the fold $F^{-}$ is a regular jump point. Thus a transition in the dynamics must happen for $a\sim0$ near the lower fold $F^{-}$ and, depending on *c*, we classify the *singular contact point*$F^{-}$ as follows:

- For $a=0$ and $c > 0$, the fold $F^{-}$ is a singular (or slow–fast) Andronov–Hopf point; see Sect. 4.
- For $a=c=0$, the fold $F^{-}$ is a singular (or slow–fast) Bogdanov–Takens point; see Sect. 6.

These two local singular bifurcation points are associated with the two different neural excitability types I and II. Note that a Bogdanov–Takens bifurcation is a codimension-2 bifurcation that includes a codimension-1 Andronov–Hopf bifurcation in its unfolding. So, we also expect to find a connection between these two bifurcations as *c* tends to zero.

#### Remark 5

Although type III neurons are not associated with any bifurcation for fixed current input, these slope detectors play an important role in identifying *dynamic* changes and producing transient responses. We refer to [2] for details and [10] where type III neurons and excitability are discussed in the context of GSPT.

From Fig. 3, we can deduce the presence of another (local) codimension-2 bifurcation, a *cusp* bifurcation [17] where two codimension-1 *saddle-node* bifurcations merge.^7^ Its approximate location can be computed easily in the normal form (15), under the condition that we discard the higher order terms $O(x^{2},xy,y^{3})$ in the $\dot{x}$ equation and the $O(y^{4})$ terms in the $\dot{y}$ equation: $(a,c) = (a_{\mathrm{cusp}}+ O(\varepsilon ),c_{\mathrm{cusp}}+ O(\varepsilon ))$ with

17 $$ a_{\mathrm{cusp}} := \frac{\sigma}{27\beta^{2}} \quad \mbox{and}\quad c_{\mathrm {cusp}}:= - \frac{\sigma}{3\beta} . $$

This indicates that for fixed $0< c< c_{\mathrm{cusp}}$ we observe three equilibrium states, two of which are located on $S_{r}$ (see Assumption 3). This has interesting consequences on the global bifurcation structure of our problem; see Sect. 5.

**Fig. 5:**
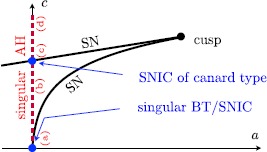
Sketch of singular limit bifurcation diagram in $(a,c)$ parameter space: singular Bogdanov–Takens and saddle-node homoclinic (*SNIC*) at the origin (*blue*); singular Andronov–Hopf branch (*red dashed*) and cusp bifurcation + saddle-node branches (*black*); see Fig. 4 for the corresponding cases (a)–(d) along the *singular AH* branch

As can be seen in Fig. 4 for $c=0$ and $I=I_{\mathrm{bif}}$, the layer problem of a type I neuron has a saddle-node bifurcation of equilibria at the lower fold $F^{-}$. This allows for the construction of a singular homoclinic orbit *Γ* as follows: we start at the saddle-node equilibrium at the lower fold $F^{-}$ and concatenate a fast fibre of the layer problem that connects to the upper stable branch $S_{a}^{+}$. Then we follow the reduced (slow) flow towards the upper fold $F^{+}$ where we concatenate a fast fibre at $F^{+}$ that connects back towards the lower attracting branch $S_{a}^{-}$. Finally, we follow the reduced (slow) flow on $S_{a}^{-}$ towards the lower fold $F^{-}$ and hence end up at the saddle-node equilibrium. This homoclinic orbit is the singular limit representation of the SNIC indicated in Fig. 1. Hence for $a=c=0$, we have identified a (global) *singular SNIC* bifurcation together with a (local) singular Bogdanov–Takens bifurcation. The unfolding of these singular bifurcations is done in Sect. 6. Figure 5 summarises all our singular limit observations.

## Type II Excitability: Singular Andronov–Hopf Bifurcation and Canard Explosion

In the case of type II excitability as shown in Fig. 3, the stable equilibrium on the lower branch $S_{a}^{-}$ crosses the lower fold $F^{-}$ at $I=I_{\mathrm{bif}}$ and moves onto the unstable middle branch $S_{r}$ where it becomes unstable as an equilibrium of the reduced problem (11). Hence, two eigenvalues change sign as we cross $F^{-}$. The bifurcation point $I_{\mathrm{bif}}$ will in general depend on *λ*; throughout this section, *λ* will be fixed and we will not further stress the dependence on *λ*, as the corresponding bifurcation is codimension-1 and only *I* is needed as a parameter for its unfolding. We introduce

18 $$ \mathcal{G}(v,I,\lambda) := g\bigl(\varphi _{I}(v),v,0, \lambda\bigr). $$

### Assumption 5

For fixed $(I,\lambda)=(I_{\mathrm{bif}},\lambda)$, the fold point $F^{-} = (w^{-},v^{-})$ with $w^{-}=\varphi _{I}(v^{-})$ is a singular contact point that undergoes a singular Andronov–Hopf bifurcation with respect to the parameter *I* at $I=I_{\mathrm{bif}}$. More precisely, we impose

19 $$\begin{aligned} \mathcal{G}\bigl(v^{-},I_{\mathrm{bif}},\lambda\bigr) = 0, \qquad \frac{\partial\mathcal{G}}{\partial v}\bigl(v^{-},I_{\mathrm{bif}},\lambda\bigr) > 0, \qquad \frac{\partial\mathcal{G}}{\partial I}\bigl(v^{-},I_{\mathrm{bif}},\lambda\bigr) \neq 0. \end{aligned}$$

Besides the singular point near $F^{-}$ occurring in this bifurcation, there are no other singular points on $S_{a}^{-}$.

Applying the normal form transformation outlined in the proof of Proposition 1 reveals that Assumption 5 amounts to imposing

$$a\vert _{I=I_{\mathrm{bif}}}=0, \qquad c>0\quad\mbox{and}\quad\frac{\partial a}{\partial I}\neq0, $$

which can easily be verified on the canonical model (1).

### Remark 6

System (1) satisfies Assumption 5 for a range of *λ* values, along a parameter curve $I=I_{\mathrm {bif}}^{\mathrm{II}}(\lambda)$.

The nature of the singular Andronov–Hopf bifurcation can easily be seen when looking at the trace and the determinant of the Jacobian of system (3) given by

20 $$ \operatorname{tr} J= \frac{\partial f}{\partial v} +\varepsilon \frac{\partial g}{\partial w} ,\qquad \det J = \varepsilon \biggl(\frac{\partial f}{\partial v}\frac {\partial g}{\partial w}- \frac{\partial f}{\partial w} \frac{\partial g}{\partial v}\biggr) . $$

Close to the fold $F^{-}$, a bifurcation of equilibria defined by $f=g=0$ happens for $0<\varepsilon \ll1$ when $\operatorname{tr} J=0$. This implies $\frac{\partial f}{\partial v}=-\varepsilon \frac{\partial g}{\partial w}=O(\varepsilon )$ and, in the singular limit, this gives the fold condition $\frac{\partial f}{\partial v}=0$. From Assumption 5 we have $\frac{\partial f}{\partial w} \frac{\partial g}{\partial v}<0$ evaluated at $g=0$ and, hence, $\det J=O(\varepsilon ) >0$. So, we are expecting a *singular Andronov–Hopf bifurcation* for $I=I_{h}$ that creates small $O(\sqrt{\varepsilon })$ amplitude limit cycles with nonzero frequencies of order $O(\sqrt {\varepsilon })$ [14, 15, 18]. Hence, the singular nature of the Andronov–Hopf bifurcation is encoded in both, amplitude and frequency. Figure 1 shows an example of a supercritical singular Andronov–Hopf bifurcation.

Note in Fig. 1 that the $O(\sqrt{\varepsilon })$ branch of the Andronov–Hopf bifurcation suddenly changes dramatically near $I=I_{c}$. This almost vertical branch marks the unfolding of *canard cycles* within an exponentially small parameter interval of the bifurcation parameter *I*. This is often referred to as a *canard explosion* [14, 15]; it provides the necessary continuous connection between the small Andronov–Hopf limit cycles and the large relaxation cycles as shown in Fig. 1. In the singular limit, canard cycles can be identified as follows: Note that the stability switch of the equilibrium in the reduced problem (11) is due to the singular nature of system (11) at $F^{-}$; a stability switch of a single equilibrium without interacting with another equilibrium in a one-dimensional regular perturbation problem is otherwise not possible. In fact, for $I=I_{\mathrm{bif}}$ there exists no equilibrium in the reduced problem (11) due to a cancellation of a simple zero. Hence, a trajectory is able to cross from $S_{a}^{-}$ to $S_{r}$ with nonzero speed which is a hallmark of a *singular canard*. One can construct singular canard cycles that are formed through concatenations of slow canard segments and fast fibres as shown in Fig. 4(d). Note that these singular canard cycles have $O(1)$ amplitude and have a frequency $O(1)$ on the order of the slow time scale. These singular canard cycles will unfold to the above mentioned canard cycles in a canard explosion. The following summarises these observations.

### Theorem 1

*In system* (4) *under Assumptions*1*–*5, *assuming the existence of only one equilibrium and for sufficiently smallε*, *a singular Andronov–Hopf bifurcation and a canard explosion occur at*

21 $$\begin{aligned} I_{h} =& I_{\mathrm{bif}} + H_{1} \varepsilon + O \bigl(\varepsilon ^{3/2}\bigr) \quad\textit{and} \end{aligned}$$

22 $$\begin{aligned} I_{c} =& I_{\mathrm{bif}} + (H_{1} + K_{1}) \varepsilon + O\bigl(\varepsilon ^{3/2}\bigr). \end{aligned}$$

*The coefficients*$H_{1}$*and*$K_{1}$*and*, *hence*, *the type of singular Andronov–Hopf bifurcation* (*super*- *or subcritical*), *can be calculated explicitly*.

*In fact*, *for the normal form* (15) *given in Proposition *1*and under the condition that*$c>0$*is sufficiently large*, *we find with respect to the bifurcation parameterathat*$a_{\mathrm{bif}}=0$, $a_{h} = H_{1a}\varepsilon + O(\varepsilon ^{3/2})$*and*$a_{c} = (H_{1a}+K_{1a})\varepsilon + O(\varepsilon ^{3/2})$, *with*

23 $$ H_{1a} = \frac{c}{2}\sigma,\qquad K_{1a} = - \frac{c}{4} \biggl(\sigma+ \frac{3}{2}\beta c \biggr). $$

*When*$K_{1a}>0$, *the Hopf bifurcation is supercritical*; *when*$K_{1a}<0$, *it is subcritical*.

### Proof

We refer to [15], where the main part of the statement is shown. Here we just restrict to computing $H_{1a}$ and $K_{1a}$ in the normal form (15).

Let us start with $H_{1a}$, given (21). A simple asymptotic analysis reveals that a singularity is located at $(x,y) = (\varepsilon ^{2}\frac{H_{1a}}{c^{2}} + O(\varepsilon ^{3}),\varepsilon \frac {H_{1a}}{c}+O(\varepsilon ^{2}) )$, about which the linearisation of the vector field has a trace given by $(-\sigma+ \frac{2H_{1a}}{c})+O(\varepsilon ^{2})$. The Hopf bifurcation hence occurs at $H_{1a}=\frac{c}{2}\sigma$.

Next we focus on the canard value $H_{1a}+K_{1a}$. Since the singular Hopf point is of generic nature, the parameter value at which canards are present are the same parameter values for which there exists a smooth asymptotic expansion $x = \varphi (y) + \varphi _{1}(y)\varepsilon + \varphi _{2}(y)\varepsilon ^{2} + O(\varepsilon ^{3})$ representing an invariant graph. Expressing the invariance by plugging the series in the differential equations yields expressions for $\varphi _{1}$ and $\varphi _{2}$, given $a_{c} = (H_{1a}+K_{1a})\varepsilon + O(\varepsilon ^{2})$. Then imposing the requirement that $\varphi _{2}$ should not have a pole at $y=0$ yields a condition on $H_{1a}+K_{1a}$ that leads to the required result. □

### Remark 7

By actively using the singular nature of canards, the above calculation also presents an alternative way to find the first Lyapunov coefficient $K_{1a}$ to determine the criticality of the singular Andronov–Hopf bifurcation.

In the singular limit, we have $I_{h}=I_{c}=I_{\mathrm{bif}}$ indicating the singular nature of the bifurcation. Note that the classic definition of type II excitability refers to the slow $O(\varepsilon )$ frequency band of the large relaxation oscillations which does not vary much (and not to the actual intermediate $O(\varepsilon ^{1/2})$ singular Andronov–Hopf bifurcation frequency).

A closer look at the expression for the first Lyapunov coefficient $K_{1a}$ in Theorem 1 indicates that there is a change of criticality at $c = c_{\mathrm{bautin}} + O(\varepsilon ^{1/2})$ with

24 $$ c_{\mathrm{bautin}} := -\frac{2\sigma}{3\beta} , $$

which evaluates to $c_{\mathrm{bautin}} = \frac{2d^{2}}{3}$ in the canonical model (1).

### Lemma 1

*For fixed*$0<\varepsilon \ll1$, *the normal form* (15) *has for*$c\approx c_{\mathrm{bautin}}$*a codimension*-2 Bautin (generalised Andronov–Hopf) *bifurcation point*.

The Bautin bifurcation [17] indicates for fixed $0< c< c_{\mathrm {bautin}}$ that we are dealing with a subcritical singular Andronov–Hopf bifurcation (under the variation of the parameter *a*) which is accompanied by another codimension-1 bifurcation, a *saddle-node of periodic orbits (SNPO)* bifurcation that has branched of the codimension-2 Bautin point. Due to the singular nature of our problem, this SNPO bifurcation is a bifurcation of canard cycles. Thus it happens exponentially close to the canard parameter value $a_{c}$ defined in Theorem 1.

## Type I/II Excitability Transition Regime: Incomplete Canard Explosion

Recall from (17) that for $\varepsilon =0$ we observe a codimension-2 cusp bifurcation at $(a,c)=(a_{\mathrm{cusp}},c_{\mathrm {cusp}})$, which persists along a parameter curve $\{(a_{\mathrm{cusp}}(\varepsilon ),c_{\mathrm {cusp}}(\varepsilon ),\varepsilon ) : \varepsilon \in[0,\varepsilon _{0}]\}$ in $(a,c,\varepsilon )$ parameter space:

### Lemma 2

*For fixed*$0<\varepsilon \ll1$, *the normal form* (15) *has for*$c\approx c_{\mathrm{cusp}}$*a codimension*-2 cusp *bifurcation point*.

This lemma indicates that the cusp has no singular nature^8^ with respect to the limit $\varepsilon \to0$. For fixed $0< c< c_{\mathrm{cusp}}$, we have three equilibrium states, at least two of which are located on $S_{r}$ (see Assumption 3). This changes the global bifurcation structure of our problem. While we still observe a singular Andronov–Hopf bifurcation with respect to the parameter *a*, the growth of the limit cycles is, however, bounded as one approaches a homoclinic connection towards one of these additional equilibria (which is of saddle type). The next theorem discusses this scenario, which describes a first transition from type II excitability towards the type I limiting situation. We formulate the results concerning the main system, but we will make the description according to the parameters introduced for the normal form (15) in Sect. 3.1. For the canonical model (1), the relation between *a* and *I* is trivial, while in general the relation between $(I,\lambda)$ and $(a,c)$ may be complicated, though Sect. 3.1 entails a procedure on how to compute the change of parameters.

### Remark 8

We denote by $c_{\mathrm{sn}}^{-}>0$ the *c*-coordinate where one of the SN branches intersects the *c*-axis (‘SNIC of canard type’ in Fig. 5). In the normal form (15) excluding the higher order (big-oh) terms, we know that $0< c_{\mathrm{sn}}^{-}< c_{\mathrm {cusp}}$. We assume that this is also the case with the big-oh terms included.

### Theorem 2

*In system* (15) *under Assumptions*1*–*5, *for fixed*$0< c< c_{\mathrm{sn}}^{-}< c_{\mathrm{cusp}}$*and*$0<\varepsilon \ll1$*there exists an unstable equilibrium on the middle branch*$S_{r,\varepsilon }$*bounded away from the lower fold*$F^{-}$. *Furthermore*, *there exist functions*

$$0 < a_{\mathrm{snpo}}(\varepsilon ) < a_{\ell}(\varepsilon ) < a_{s}(\varepsilon ) < a_{c}(\varepsilon ) < a_{h}(\varepsilon ) < a_{\mathrm{sn}}^{+}(\varepsilon ) $$

**Fig. 6:**
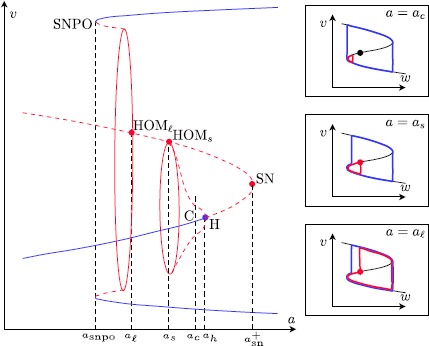
Bifurcation diagram for fixed $0< c< c_{\mathrm{cusp}}$. *The parabola* shows the two equilibra near the fold $F^{-}$ (the third equilibrium branch and second SN are not shown). The *a*-*axis* is not shown on scale, as the distance between $a_{\mathrm {snpo}}$ and $a_{c}$ should be exponentially small. Keeping that in mind, an incomplete canard explosion is seen as *a* goes from $a_{c}$ to $a_{s}$

*that all converge to zero in the singular limit*$\varepsilon \to0$ (*except*$a_{\mathrm{sn}}^{+}$) *and for which the following holds* (*see also Fig*. 6):

1. *For*$a_{\mathrm{sn}}^{+}< a$, *the fold*$F^{-}$*is of regular jump type and a large stable relaxation cycle exists*.
2. *At*$a=a_{\mathrm{sn}}^{+}$, *a saddle*-*node bifurcation of singular points on the middle branch*$S_{r,\varepsilon }$*in an*$O(c)$-*neighbourhood of*$F^{-}$; *the large relaxation cycle persists*.
3. *For*$a_{h} < a < a_{\mathrm{sn}}^{+}$, *the system has a saddle*$p_{+}$*and an unstable focus*/*node*$p_{-}$*on the middle branch*$S_{r,\varepsilon }$*surrounded by the large relaxation cycle*. *The unstable focus*/*node*$p_{-}$*is closer to the fold*$F^{-}$.
4. *At*$a=a_{h}$, $p_{-}$*changes stability and a subcritical singular Andronov–Hopf bifurcation takes place*; *the large relaxation cycle persists*.
5. *For*$a_{c}< a< a_{h}$, *repelling small*-*amplitude limit cycles appear around the stable focus*$p_{-}$; *the large relaxation cycle persists*.
6. *For*$a_{s}< a< a_{c}$, *small jump*-*back canard cycles appear that rapidly grow in amplitude* (*canard explosion*); *the large relaxation cycle perturbs to a large*-*amplitude jump*-*forward canard cycle*.
7. *At*$a=a_{s}$, *a small jump*-*back homoclinic loop of canard type*, *issued from the saddle*$p_{+}$, *appears together with a stable large*-*amplitude canard cycle*.
8. *For*$a_{\ell} < a < a_{s}$, *the small homoclinic loop breaks and only the stable large*-*amplitude canard cycle persists*.
9. *At*$a=a_{\ell}$, *a large*-*amplitude homoclinic loop of canard type*, *issued from the saddle*$p_{+}$, *appears together with the outer large*-*amplitude cycle*.
10. *Asadecreases from*$a_{\ell}$, *large*-*amplitude canard cycles appear that grow in amplitude until it disappears in a saddle*-*node bifurcation of limit cycles at*$a=a_{\mathrm{snpo}}$.

### Proof

Before proving this theorem, we would like to mention that the distance between $a_{h}$ and $a_{c}$ is $O(\varepsilon )$, just like in the previous singular Andronov–Hopf case, while the values $a_{c}$, $a_{s}$, $a_{\ell}$ and $a_{\mathrm{snpo}}$ are all exponentially close to each other.

*Singularities near the fold*. For fixed $0< c< c_{\mathrm{cusp}}$, the singularities are found at intersections of the nullclines $cy-a-x+O(x^{2},y^{3},xy)=0$ and $y^{2}-x+\beta y^{3} + O(y^{4})=0$, hence have *y*-coordinates satisfying $\rho(y) := -a+cy-y^{2}+O(y^{3})=0$. The value $a_{\mathrm{sn}}^{+}$ corresponds to the solution of $\{\rho(y)=0,\rho'(y)=0\}$, solved w.r.t. $(y,a)$. It yields

25 $$ a_{\mathrm{sn}}^{+} = \frac{c^{2}}{4} + O\bigl(c^{3}\bigr). $$

At $a=a_{\mathrm{sn}}^{+}$, the double singular point appears (in the singular limit $\varepsilon =0$) at $y=\frac{c}{2} + O(c^{2})$, i.e. on the middle branch, and for $a_{h}< a< a_{\mathrm{sn}}^{+}$ there are two singular points: one unstable node/focus $p_{-}$ close to the fold, and a saddle $p_{+}$ that is $O(c)$-away from the fold, both located on the middle branch.

For $a>a_{h}$, the location of the singularities implies that the fold $F^{-}$ is a jump point and a stable relaxation cycle is present. This shows parts (1)–(3).

### Remark 9

Note, there exists also a third equilibrium on the middle branch, an unstable node/focus denoted by *n*, bounded away from the fold $F^{-}$. This third equilibrium bifurcates with $p_{+}$ along the second saddle-node branch (see Fig. 5).

*Singular Andronov–Hopf bifurcation*. As demonstrated in the proof of Theorem 1, we have a singular Andronov–Hopf bifurcation

$$a_{h} = \varepsilon \frac{c}{2} + O\bigl(\varepsilon ^{3/2}\bigr), $$

and the criticality depends on the sign of $K_{1a} = -\frac{c}{4}(1 + \frac{3}{2}\beta c) = -\frac{c}{4}(1 + O(c))$ which is negative since $0< c< c_{\mathrm{cusp}}< c_{\mathrm{bautin}}$, i.e. the Andronov–Hopf bifurcation is subcritical. This shows parts (4)–(5).

### Remark 10

The canard parameter value $a_{c}$ is not strictly defined, as also a “small-amplitude limit cycle” is not strictly defined. We choose a *δ*-neighbourhood of the fold and the moment where the canard cycles grow out of this *δ*-neighbourhood along the canard explosion, we define the parameter value $a=a_{c}(\varepsilon )$. (In other words, $a_{c}$ lies beyond the “birth of canards”.)

*Incomplete canard explosion and homoclinic saddle loops*. The presence of the saddle $p_{+}$ shows that the canard cycles cannot grow unlimitedly during the canard explosion. We give here a short overview of the proof of the presence of small canard cycles because we will use elements of the proof to show the existence of canard homoclinics.

For fixed $c>0$, we rescale $a=\sqrt{\varepsilon }A$, thus studying an $O(\sqrt{\varepsilon })$ neighbourhood of the positive *c*-axis in $(a,c)$ parameter space along which we observe a singular Andronov–Hopf bifurcation; see Fig. 5. We consider, in normal form coordinates, two transverse sections: a section $S = \{ y=0, x_{0}< x< x_{1}\}$ which is transverse to the fast flow and a section $T = \{ y=0,\vert x\vert =O(\varepsilon )\}$ close to the singular fold $F^{-}$. Using geometric singular perturbation theory, we find that both the forward flow and the backward flow of the vector field takes points of *S* to *T*; indeed, the forward resp. backward fast flow takes points of *S* to $S_{a}^{-}$ resp. $S_{r}$, after which the dynamics of the slow flow governs the drift towards *T*. It defines two maps

26 $$ x_{T} = \varepsilon F(x_{S},\sqrt{\varepsilon },A),\qquad x_{T} = \varepsilon B(x_{S},\sqrt {\varepsilon },A), $$

where the forward map *F* and the backward map *B* are known to be smooth in terms of $(x_{S},\sqrt{\varepsilon },A)$; see [19]. To be more precise, let $x_{S}^{+}$ be the (parameter dependent) coordinate of the intersection of *S* with the fast fibre separatrix of the saddle $p_{+}$. Then this coordinate is the supremum of the *x*-coordinates for which the backward map in (26) is defined. In [19] it is then shown that $F-B=0$ at $(\sqrt{\varepsilon },A)=(0,0)$ (independently of $x_{S}$), and $\frac{\partial}{\partial A}(F-B)\neq0$ at that point. Application of the implicit function theorem leads to the presence of a canard curve $A=\sqrt{\varepsilon } A_{\mathrm{canard}}(x_{S},\sqrt{\varepsilon })$ along which the vector field has a canard periodic orbit that intersects the section *S* at *x*-coordinate $x_{S}$. The canard curve $A_{\mathrm{canard}}$ is smooth in *ε* and its value depends in an exponentially small way on $x_{S}$. In other words, canard cycles are found in an exponentially small wedge along an $O(\varepsilon )$ neighbourhood of the positive c-axis in $(a,c)$-parameter space.

We can further rely on the results in [20], where it is shown that the maps (26) are smooth up to and including (at its extension) the boundary $x=x_{S}^{+}$. This implies that the canard value $a_{s}=\varepsilon A_{\mathrm{canard}}(x_{S}^{+},\sqrt{\varepsilon })$ obtained above is actually a parameter curve along which the vector field has a homoclinic saddle loop of canard type (of ‘jump-back’ type). This proves part (7) of the theorem.

### Remark 11

Around the unstable canard cycle (or homoclinic a bit later on), there appears a big relaxation oscillation of canard type. It lies close to the full relaxation oscillation, but travels an $O(c)$-distance along the middle repelling branch (see Fig. 6). The exact distance travelled along the middle branch can be computed using slow-divergence integrals and exit–entry relations; we refer to the literature [21].

By introducing an alternative section $\tilde{S}$ between the middle and upper branch instead of *S*, we can treat homoclinic saddle loops of the ‘jump-away’ type in a completely similar way. The only thing that changes is that points of $\tilde{S}$ undergo a large-amplitude oscillation in their way to *T* in positive time (travelling along $S_{a}^{+}$ towards the jump point $F^{+}$, jumping off towards $S_{a}^{-}$). The smoothness of the transition maps and the application of the implicit function theorem is analogous. This defines $a=a_{\ell}< a_{s}$ and proves part (9) of the theorem.

For $a< a_{\ell}(\varepsilon )$, the homoclinic connection breaks into a repelling large-amplitude cycle. While *a* proceeds to the outside of an $O(\varepsilon )$-neighbourhood of 0, it encounters the relaxation-like attracting cycle that surrounded all repelling cycles. They disappear in a saddle-node bifurcation of limit cycles at $a=a_{\mathrm{snpo}}< a_{\ell}$. This proves part (10) of the theorem.

**Fig. 7:**
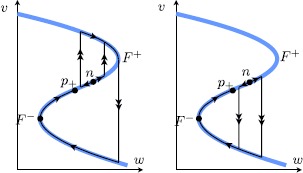
Heteroclinic connections of canard type undergo a transition from headless canard to canard with head, from the jump-back canard homoclinic to the jump-away canard homoclinic

For $a_{\ell}< a< a_{s}$ in between the two homoclinic curves, the stable manifold $W^{s}$ connects in reverse time to an orbit that follows the large homoclinic for some time, but exiting at a time prior to the time needed to connect back to $p_{+}$. Therefore, no limit cycles may appear. In fact, since we know there is an additional singularity on the middle branch $S_{r}$, denoted by *n*, and assuming it is of node type, then the exponential gap between the two homoclinics is filled by canard curves along which canard-type heteroclinic connections appear between *n* and $p_{+}$; see Fig. 7. The transition from headless heteroclinic canard to heteroclinic canard with head can be seen as a natural continuation of the truncated canard explosion of the canard homoclinics. The proof of the presence of such heteroclinics is completely analogous to above. In particular, no limit cycles are present in this scenario. This proves part (8) and finishes the proof of the theorem. □

### Termination of Homoclinic Saddle Loops: SNICs

In the canonical form (1), the singular Andronov–Hopf curve intersects the saddle-node bifurcation curve along which the saddle $p_{+}$ collides with a third singularity, a node *n* on the middle branch $S_{r}$. Expressing (1) in the local coordinates (14), this singular bifurcation point is marked in blue in Fig. 5 and has coordinates $(a_{\mathrm{sn}}^{-},c_{\mathrm{sn}}^{-})=(0,-\frac{1}{4\beta})$. In this section, we discuss how this codimension-2 singular bifurcation point perturbs to $\varepsilon >0$.

The SN-curve perturbs regularly for positive values of *ε* to a curve $c = c_{\mathrm{sn}}(a,\varepsilon )$. Observe that the part of the AH curve between the origin and this codimension-2 bifurcation point perturbs to a wedge of canard curves, corresponding to the incomplete canard explosion discussed in Theorem 2.

As in the case of the saddle homoclinics, denote by $x_{S}^{+}$ the intersection of the section *S* with the unstable separatrix of the singularity $p_{+}$, up to the point where it becomes a saddle-node. Then the part $\{x< x_{S}^{+}\}$ of *S* is the part for which the backward map $B\colon S\to T$ is well defined. Applying the results in [20], we know that the map *B* has a $C^{k}$-smooth extension (for any *k*) to the boundary of its definition domain. It implies that we can define $B(x_{S}^{+},\varepsilon ,a,c_{\mathrm{sn}}(a,\varepsilon ))$ and also $F(x_{S}^{+},\varepsilon ,a,c_{\mathrm{sn}}(a,\varepsilon ))$, like in (26) but where we made the dependence of *c* explicit. Therefore, solving $F-B=0$ using the implicit function theorem with respect to the rescaled parameter *A* (recall $a=\sqrt{\varepsilon }A$) allows us to prove the presence of saddle-node homoclinics of (‘jump-back’) canard type.

As before, replacing the section *S* with the alternative $\tilde{S}$, we can apply the same reasoning to prove the presence of a canard value along which there is a saddle-node homoclinic of (‘jump-away’) canard type. Since the canard values $A_{\mathrm{canard}}$ are smooth in terms of $x_{S}$, we clearly see that the homoclinic loop curves (of jump-back and jump-away type) terminate at corresponding saddle-node homoclinic at the saddle-node bifurcation curve.

The exponential wedge on the SN-curve between the two terminal points are terminal points of SNIC canard curves that correspond to heteroclinic canards as shown in Fig. 7. Visually, it is clear how in Fig. 7, a heteroclinic connection tends towards a SNIC as *n* and $p_{+}$ approach each other in a SN bifurcation. The method of proof is similar to the one exposed before.

#### Remark 12

In the case $c_{\mathrm{sn}}^{-}< c< c_{\mathrm{cusp}}$ fixed, the second SN-bifurcation is located ahead of the singular (subcritical) AH-bifurcation and we observe a complete canard explosion (including a SNPO bifurcation of canard cycles).

In the case $c_{\mathrm{cusp}}< c< c_{\mathrm{bautin}}$ fixed, there is only the singular (subcritical) AH-bifurcation and we also observe a complete canard explosion (including a SNPO bifurcation of canard cycles).

**Fig. 8:**
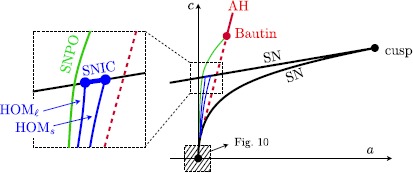
Sketch of bifurcation diagram in $(a,c)$ parameter space for $0<\varepsilon \ll1$: cusp bifurcation and *SN* branches (*black*); *Bautin* bifurcation point (*red*) with Andronov–Hopf branches (sub = *dashed*/super = *solid*); and saddle-node of periodic orbit (*SNPO*) branch (*green*); small and large homoclinic ($\mathit{HOM}_{s}$ and $\mathit{HOM}_{\ell}$) branches (*blue*); *SNIC* segment (*blue*) on the *SN* branch $a_{\mathrm{sn}}^{-}$

Figure 8 summarises all our observations for $0<\varepsilon \ll1$ (compare with the singular limit bifurcation diagram in Fig. 5).

## Type I Excitability: Singular Bogdanov–Takens/SNIC Bifurcation

In this section, we discuss the origin $(a,c)=(0,0)$ which represents a local singular Bogdanov–Takens and a global singular SNIC bifurcation point; see Fig. 5. Let us first formulate general conditions under which the origin in the $(a,c)$-diagram of the local normal form is relevant for system (3) under the condition that Assumptions 1–4 are satisfied. We look at the lower fold point $F^{-}$ when it violates Assumption 5, i.e. we are interested in parameter values $\lambda=\lambda_{\mathrm{bif}}$ where

$$\frac{\partial\mathcal{G}}{\partial v}\bigl(v^{-},I_{\mathrm{bif}},\lambda _{\mathrm{bif}}\bigr) = 0 , $$

with $\mathcal{G}$ defined in (18). This condition is typically violated in 1-parameter families. For SNIC bifurcations to appear with a saddle-node near the fold point $F^{-}$, we will hence impose the following condition.

### Assumption 6

For fixed $(I,\lambda)=(I_{\mathrm{bif}},\lambda_{\mathrm{bif}})$, the fold point $F^{-} = (w^{-},v^{-})$ is a singular contact point that undergoes a singular Bogdanov–Takens bifurcation with respect to the parameters $(I,\lambda)$ at $(I,\lambda)=(I_{\mathrm{bif}},\lambda_{\mathrm{bif}})$. More precisely, we impose (on top of Assumptions 1–4):

27 $$\begin{gathered} \begin{aligned} \mathcal{G}\bigl(v^{-},I_{\mathrm{bif}},\lambda_{\mathrm{bif}}\bigr) &= 0,\\ \frac{\partial\mathcal{G}}{\partial v}\bigl(v^{-},I_{\mathrm{bif}},\lambda _{\mathrm{bif}} \bigr) &= 0,\qquad \frac{\partial^{2} \mathcal{G}}{\partial v^{2}}\bigl(v^{-},I_{\mathrm{bif}},\lambda _{\mathrm{bif}}\bigr) > 0, \end{aligned} \end{gathered}$$

28 $$\begin{gathered} \begin{aligned} \frac{\partial\mathcal{G}}{\partial I}\bigl(v^{-},I_{\mathrm{bif}},\lambda _{\mathrm{bif}}\bigr)&\neq0, \\ \frac{\partial\mathcal{G}}{\partial\lambda}\bigl(v^{-},I_{\mathrm {bif}}, \lambda_{\mathrm{bif}}\bigr)&=0,\qquad \frac{\partial^{2}\mathcal{G}}{\partial\lambda\partial v}\bigl(v^{-},I_{\mathrm {bif}}, \lambda_{\mathrm{bif}}\bigr)\neq0. \end{aligned} \end{gathered}$$

Besides the possible singular points near $F^{-}$ occurring in this bifurcation, there are no other singular points on $S_{a}^{-}$.

Conditions (27) imply that the fold point $F^{-}$ is a local codimension-2 singular point. Conditions (28) imply that a complete unfolding of the singularity is obtained upon varying $(I,\lambda)$. In fact, the conditions in (28) could be replaced by the slightly more general condition,

$$\det\frac{\partial(\mathcal{G},\mathcal{G}_{v})}{\partial(I,\lambda )}\bigl(v^{-},I_{\mathrm{bif}},\lambda_{\mathrm{bif}}\bigr) \neq0 $$

where $\mathcal{G}_{v}:= \partial\mathcal{G}/\partial v$, but we prefer to keep (28) in order to be able to identify *I* as the Hopf breaking parameter among the two parameters.

### Remark 13

It can be seen that conditions (27) and (28) imply the following conditions on the normal form (15):

$$\begin{aligned} (a,c)\vert _{(I,\lambda)} =&(I_{\mathrm{bif}},\lambda_{\mathrm{bif}})=(0,0), \qquad\sigma= +1, \\ \frac{\partial a}{\partial I} \neq&0, \qquad \frac{\partial a}{\partial \lambda} = 0,\qquad \frac{\partial c}{\partial\lambda}\neq0, \end{aligned}$$

which are verified on the canonical model (1).

Under these conditions it is well known that in *ε*-dependent rescaled coordinates, a regular Bogdanov–Takens bifurcation takes place; see [16]. As a consequence, the presence of small-amplitude homoclinics is clear in some parameter subset. Furthermore, as (singular) Andronov–Hopf bifurcations form part of the bifurcation diagram, canard-type orbits are present. Indeed, the double singularity in the slow dynamics at $x=0$ may unfold in a way that the fold point becomes a canard point and an extra saddle-singularity in the slow dynamics on the middle branch may appear. In that way, an incomplete canard explosion can be observed that terminates in a canard-type saddle homoclinic (‘jump-back’, without ‘head’). In fact, these are phenomena that appear locally near the Bogdanov–Takens fold point.

Besides the small-amplitude phenomena near the Bogdanov–Takens point, we consider orbits that are close to the singular saddle-node homoclinic loop *Γ* shown in Fig. 4. We expect the existence of large-amplitude saddle-node homoclinics (SNICs) and as in the previous section, we also expect large-amplitude saddle homoclinics as well as relaxation oscillations.

In order to get a hold on the parameters close to $c=0$, we rescale the parameters and introduce

29 $$ (c,a) = \bigl(\varepsilon C,\varepsilon ^{2}A\bigr),\quad(C,A) \in[0,M]\times[-M,M] $$

for some large $M>0$. By doing this we in fact assume that $c=O(\varepsilon )$ and $a=O(\varepsilon ^{2})$. After the parameter rescaling (29), we study the system

30 $$ \begin{aligned} x' &= \varepsilon \bigl(- \varepsilon ^{2}A + \varepsilon Cy - x + O\bigl(x^{2},y^{3},xy,\varepsilon y^{2}\bigr) \bigr), \\ y' &= y^{2}-x + O\bigl(y^{3}\bigr) . \end{aligned} $$

The singularity at $(x,y,\varepsilon )=(0,0,0)$ has been described in [16] as a *slow–fast Bogdanov–Takens point*.^9^ In that paper, it is shown that a BT bifurcation takes place near the origin. More importantly, it is shown that the phase portraits associated with the BT bifurcation are the only phase portraits seen in a small neighbourhood of the origin. The paper does not deal with any interactions with global return mechanisms, i.e. the interaction of the (local) singular BT and the (global) singular SNIC have not been studied. We will therefore repeat part of the local analysis, with the focus on the interaction with the global return mechanism.

### Blow-up of the Singular Fold

Near the fold, we study the system using *blow-up* [14, 15]. We write

$$(x,y,\varepsilon ) = \bigl(r^{2}X,rY,rE\bigr),\quad r\geq0, (X,Y,E)\in S^{2}_{+}, $$

where $S^{2}_{+}$ denotes the half-sphere $X^{2}+Y^{2}+E^{2}=1$ with $E\geq0$ (also known as *Poincaré or blow-up sphere*). The weights are chosen in a way that the higher order (big-oh) terms in (30) have also higher order in the rescaled equation.

As is usual in geometric desingularisation, we study the flow on the half-sphere in different (coordinate) charts. Two charts are important: the chart $K_{1}$ (or the phase-directional rescaling chart), and the chart $K_{2}$ (or the family rescaling chart). The $K_{1}$ chart is used to extend the orbits along the slow manifold (which are directed towards the fold) to a neighbourhood that is at distance $O(\varepsilon )$ from the origin. While this transition is the most technical and least obvious for the reader who is not accustomed to the blow-up method, it fortunately is that part where the study of system (30) agrees with the results in studies of a classical regular jump point. Hence, we do not present detailed computations in the chart $K_{1}$, but focus on presenting important facts and refer to the literature [14, 15] for a detailed analysis.

**Fig. 9:**
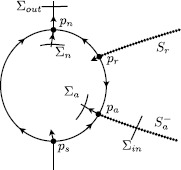
Blow-up of the singular fold. ‘Birds-eye view’ of the upper blown-up sphere $X^{2}+Y^{2}+E^{2}=1$ in $(X,Y)$-space. Normal hyperbolicity of the manifolds $S_{a}$ and $S_{r}$ is gained at the equator, allowing one to extend them onto the blown-up sphere near singularities $p_{a}$, respectively, $p_{r}$. The two additional singularities represent connection to the fast fibre at $F^{-}$

*The phase-directional rescaling chart*$K_{1}$. Here, we explain the dynamics near the equator of the blow-up sphere $S^{2}_{+}$. When presenting a picture in blown-up $(x,y,\varepsilon )$-space, where the origin is replaced by (or *blown-up* to) a sphere, we can position the point of view from the top of the *ε*-axis; looking down on the $(X,Y)$-plane we see the spherical surface $X^{2}+Y^{2}+E^{2}=1$ with $E\geq0$ as the interior of a circle $X^{2}+Y^{2}=1$, and the equator as the circle with the outer slow–fast dynamics around it; see Fig. 9.

Calculations in $K_{1}$ reveal two hyperbolic saddle singularities, $p_{s}$ and $p_{n}$, and two semi-hyperbolic singularities $p_{a}$ and $p_{r}$ along the equator. Combining information from the slow–fast dynamics near $F^{-}$ with information obtained in chart $K_{1}$ allows one to reconstruct the dynamics near the circle shown in Fig. 9.

Combining the global return mechanism, which defines a map $\varSigma _{\mathrm{out}}\to\varSigma_{\mathrm{in}}$, with the information from this chart allows one to show the smoothness and exponentially contractiveness of the $(\varepsilon ,C,A)$-family of maps

31 $$ \varSigma_{n}\to\varSigma_{a}. $$

Given the uniqueness of the centre separatrix issued from $p_{a}$, one can prove that the image of any small section $\varSigma_{n}$ under this map limits to this centre separatrix (intersected with $\varSigma_{a}$) as $\varepsilon \to0$. To characterise the global dynamics, it is therefore important to know the dynamics of this centre separatrix. In particular, a connection of $\varSigma_{a}$ to $\varSigma_{n}$ will distinguish whether or not singular points are met, or whether a regular ‘jump point-like’ connection is possible. This study will be done in the family directional rescaling chart.

*The family rescaling chart*$K_{2}$. Once the orbits have passed the chart $K_{1}$, we can assume that $x=O(\varepsilon ^{2})$ and $y=O(\varepsilon )$. In the blow-up coordinates, this means that $(X,Y,E)$ is bounded away from the equator $\{E=0\}$ of the sphere (since then it means $\varepsilon \sim r$). It is well known that a study of that part of the sphere can be established by looking at an *ε*-dependent rescaling

32 $$ (x,y) = \bigl(\varepsilon ^{2}X,\varepsilon Y\bigr),\quad(X,Y) \in[-R,R]^{2} $$

for some large $R>0$. Applying this rescaling to (30), we can divide out a common factor *ε*, thus transforming the system into a regular perturbation family^10^

33 $$ \begin{aligned} \dot{X} &= -A + CY - X + O(\varepsilon ), \\ \dot{Y} &= Y^{2}-X + O(\varepsilon ). \end{aligned} $$

### Local and Global Codimension-2 Bifurcations

System (33) describes the flow in the interior of the sphere as shown in, e.g., Fig. 9, and it can be analysed by means of classic bifurcation analysis. Together with the information obtained from the global return mechanism, we are able to describe all observed local and global bifurcations in $(A,C)$ parameter space.

*Bogdanov–Takens bifurcation*.

#### Lemma 3

*For*$\varepsilon =0$, *system* (33) *undergoes a subcritical Andronov–Hopf bifurcation when*$A=A_{h}(C)= -\frac{1}{4} +\frac{1}{2} C$, $C>1$, *and a saddle*-*node bifurcation of singularities when*$A=A_{\mathrm{sn}}^{+}(C)=C^{2}/4$. *Both bifurcation curves meet in a Bogdanov–Takens bifurcation point at*$(A,C)=(1/4,1)$. *Both bifurcations persist for*$\varepsilon > 0$.

#### Proof

There are two singular points on $X=Y^{2}$, located at $Y=Y_{\pm} := \frac{1}{2}(C \pm\sqrt{C^{2}-4A})$. The singularity at $Y=Y_{+}$, denoted $p_{+}$, is always a saddle. The singularity at $Y=Y_{-}$, denoted $p_{-}$ is of focus/node type for $C>1$ and undergoes a change in the sign of the trace along $A_{h}=-\frac{1}{4}+\frac{1}{2}C$, which indicates an Andronov–Hopf bifurcation. Along this parameter line, $p_{-}$ is weakly unstable; a basic calculation shows that the first Lyapunov coefficient is positive. Hence the Andronov–Hopf bifurcation is subcritical.

The two singular points $p_{+}$ and $p_{-}$ collide along $A_{\mathrm{sn}}^{+}=C^{2}/4$ indicating a saddle-node bifurcation at $p_{\pm}$. □

#### Remark 14

**Fig. 10:**
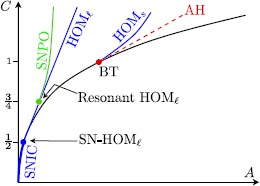
Bifurcations found in (33) for $\varepsilon =0$ in the $(C,A)$-parameter plane. Codimension-2: Bogdanov–Takens (*BT*), resonant homoclinic (*Resonant* $\mathit{HOM}_{\ell}$) and saddle-node homoclinic ($\mathit{SN}\mbox{-}\mathit{HOM}_{\ell}$); codimension-1: saddle-node (*SN*), Andronov–Hopf (*AH*), saddle homoclinic ($\mathit{HOM}_{s}$ and $\mathit{HOM}_{\ell}$), saddle-node of limit cycles (*SNPO*)

Let us mention, without proof, that the homoclinic saddle-loop bifurcation curve ($\mathrm{HOM}_{s}$ in Fig. 10) of the Bogdanov–Takens point (BT) at $(A,C)=(\frac{1}{4},1)$ lies between the Andronov–Hopf curve (AH) and the parameter line $A=A_{\ell}(C)=-\frac {1}{16}+\frac{C}{4}$ ($\mathrm{HOM}_{\ell}$) and tends towards this parameter line as it approaches infinity; see Fig. 10. This can be seen by studying (33) for $\varepsilon =0$ near infinity [22].

*Saddle-node homoclinic bifurcation*. The following proposition states some properties of system (33) for $\varepsilon =0$. As mentioned before, we focus on the interaction of local dynamics with the global return mechanism (31). In particular, we want to understand the dynamics of the centre separatrix of $p_{a}$.

#### Proposition 2

**Fig. 11:**
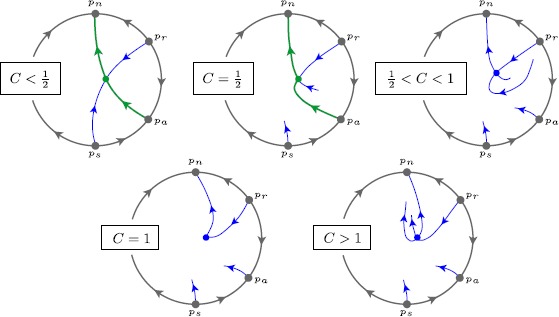
Behaviour of (33) on the Poincaré disc for $\varepsilon =0$, along the SN-curve $A_{\mathrm{sn}}^{+}(C)=\frac{C^{2}}{4}$. For $C\le \frac{1}{2}$, the point $p_{a}$ connects to the SN point $p_{\pm}$. At $C=1$, a BT point $p_{\pm}$ occurs, not connected to $p_{a}$, however

*Along the saddle*-*node bifurcation line*$A_{\mathrm{sn}}^{+}(C)=C^{2}/4$, *we have the following behaviour of* (33) *for*$\varepsilon =0$ (*see Fig*. 11):

1. *When*$C<\frac{1}{2}$, *the separatrix coming from*$p_{a}$*connects to a centre*-*stable separatrix of the saddle*-*node singularity*$p_{\pm}$. *The unique unstable centre separatrix of*$p_{\pm}$*connects to*$p_{n}$.
2. *When*$C=\frac{1}{2}$, *the separatrix coming from*$p_{a}$*connects to the hyperbolic attracting separatrix of the saddle*-*node singularity*$p_{\pm}$. *The unique unstable centre separatrix of*$p_{\pm}$*connects to*$p_{n}$.
3. *When*$C > \frac{1}{2}$, *the separatrix coming from*$p_{a}$*connects directly to*$p_{n}$*along a regular orbit*. *This is the jump scenario*. *In particular*, *the BT point*$p_{\pm}$ ($C=1$) *is not connected to the separatrix*.
4. *The attracting separatrix of the saddle*-*node point*$p_{\pm}$*and the separatrix coming from*$p_{a}$*break regularly with respect to the parameter C*.

#### Proof

The proof uses basics from planar theory of vector fields (e.g. invariant curves, isoclines, positive invariant sets). It requires some computations, but as it concerns basic properties we have left the details for the reader.

For $C<1/2$, we define $W = X-Y^{2} + (C-1)Y - \frac{1}{2}(C-1)C$. Notice that the saddle-node singularity $p_{\pm}$ is a point on the parabola $W=0$ and that $\dot{W}\vert _{W=0} = (1-2C)(Y-\frac{C}{2})^{2}$ which is positive except at the SN point $p_{\pm}$. Using information from infinity (i.e. from chart $K_{1}$), we see that the separatrix from $p_{a}$ enters the region $\{W>0\}$ which is positively invariant. Hence the *ω*-limit set of the separatrix has to be the vertex of the parabola. Finally, the hyperbolic separatrix of the saddle-node singularity $p_{\pm}$ is tangent to $\partial\{W=0\}$ which implies it lies outside the positive invariant set $\{W>0\}$. This proves part (1).

For $C=\frac{1}{2}$, the singularity $p_{\pm}$ lies on the invariant parabola from Lemma 3, which then coincides with the separatrix coming from $p_{a}$. It is not hard to verify that the hyperbolic eigenspace of the saddle-node singularity $p_{\pm}$ coincides with the tangent space of the parabola. This proves part (2).

For $C>\frac{1}{2}$, we define $V= X-Y^{2}-\frac{1}{2}Y-(\frac{1}{8}-\frac{C}{2})$. One can verify that $\dot{V}\vert _{V=0} = -\frac{1}{16}(2C-1)^{2}<0$, so that $\{ V\leq0\}$ is a positive invariant set. It is a symbolic computation to verify that the separatrix coming from $p_{a}$ enters this invariant set, and hence cannot leave.^11^ On the other hand, *V* computed at the saddle-node point $p_{\pm}=(\frac{1}{4} C^{2},\frac{1}{2} C)$ yields $\frac{1}{4}(C-\frac{1}{2})>0$. We conclude that the separatrix from $p_{a}$ cannot reach $p_{\pm}$. Since $p_{n}$ is the only other option for a *ω*-limit, it shows part (3).

As for the regular breaking of the connection in part (4): we compute the stable separatrix of the saddle-node $p_{\pm}$ and compare it with the separatrix coming from $p_{a}$. For the comparison we choose an arbitrary section crossing $\{V=0\}$ and parameterise it by the levels of *V*. It is not hard to see that the separatrix coming from $p_{a}$ intersects any such section at *V*-values that are $O(C-\frac{1}{2})^{2}$. On the other hand, a variational computation of the stable separatrix of $p_{\pm}$ reveals that it is given by $V = \frac{1}{4}e^{1-4Y} (C-\frac{1}{2}) + O(C-\frac{1}{2})^{2}$. Since $\frac{1}{4}e^{1-4Y}\neq0$, it explains the transversality. This finishes the proof of the theorem. □

Clearly, the saddle-node curve $A_{\mathrm{sn}}^{+}(C)=C^{2}/4$ persists within a manifold $A_{\mathrm{sn}}^{+}(C,\varepsilon )=C^{2}/4 + O(\varepsilon )$. The regular breaking property formulated in the proposition ensures that the results persist for $\varepsilon >0$.

#### Theorem 3

*There exists a parameter surface*$A_{\mathrm{sn}}^{+}(C,\varepsilon )=C^{2}/4 + O(\varepsilon )$*along which a saddle*-*node singularity*$p_{\pm}$*exists*. *On this surface*, *there exists a curve*$C=\frac{1}{2} + O(\varepsilon )$*along which a saddle*-*node homoclinic* ($\mathit{SN}\mbox{-}\mathit{HOM}_{\ell}$) *connection appears containing the hyperbolic separatrix of the saddle*-*node*. *For*$C<\frac{1}{2} + O(\varepsilon )$*on this parameter surface*, *there is a SNIC connection containing a centre separatrix of the saddle*-*node*. *For*$C>\frac{1}{2} + O(\varepsilon )$, *there is no SNIC connection*.

#### Proof

Restrict to the saddle-node surface. Let $\gamma_{C,\varepsilon }$ be the unstable separatrix of the saddle-node $p_{\pm}$ that connects to $p_{n}$. It smoothly intersects in a point $P_{C,\varepsilon }$ the section $\varSigma_{n}$. The global return mechanism (31) takes this point to a point $Q_{C,\varepsilon }$ on $\varSigma_{a}$, where $Q_{C,0}$ lies on the centre separatrix. On the other hand, let $\nu_{C,\varepsilon }$ be the hyperbolic stable separatrix of the saddle-node $p_{\pm}$ that intersects $\varSigma_{a}$ at a point $R_{C,\varepsilon }$. From Proposition 2, we know that $Q_{\frac{1}{2},0}=R_{\frac{1}{2},0}$, and, parameterizing the section $\varSigma _{a}$ by a regular coordinate *θ*, we also know that $\frac{\partial }{\partial C}(Q_{C,0}-R_{C,0})\neq0$ at $C=\frac{1}{2}$. Hence, we can apply the implicit function theorem to prove the presence of a curve $C=\frac{1}{2}+O(\varepsilon )$ along which both points coincide and a saddle-node homoclinic connection appears. The rest of the statements follow easily from the properties at the singular limit. □

*Resonant homoclinic bifurcation*.

#### Proposition 3

**Fig. 12:**
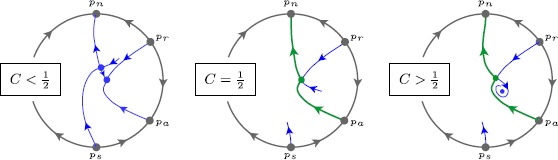
Behaviour of (33) on the Poincaré disc for $\varepsilon =0$, along the curve $A_{\ell}(C)=-\frac{1}{16}+\frac{C}{4}$. At $C=\frac{1}{2}$, the point $p_{a}$ connects to the SN point $p_{\pm}$. For $C<\frac{1}{2}$ the $p_{a}$ connects to the node $p_{-}$, for $C>\frac{1}{2}$, there is a sequence of two heteroclinic connections from $p_{a}$ to $p_{n}$, via the saddle $p_{+}$, which is resonant at $C=\frac{3}{4}$

*Along*$A_{\ell}(C)=-\frac{1}{16}+\frac{C}{4}$, *we have the following behaviour of* (33) *for*$\varepsilon =0$; *see Fig*. 12:

1. *When*$C<\frac{1}{2}$, *the centre separatrix of*$p_{a}$*connects to the node*$p_{-}$.
2. *When*$C=\frac{1}{2}$, *a SN*-*bifurcation takes place* (*see Proposition *2).
3. *When*$C>\frac{1}{2}$, *a centre separatrix of*$p_{a}$*connects to the hyperbolic saddle*$p_{+}$, *and one of unstable separatrices of the saddle connects to*$p_{n}$. *The ratio of eigenvalues is given by*$\rho(C) := 2-4C < 0$, *and the saddle is strongly resonant at*$C=\frac{3}{4}$.
4. *For any given*$C>\frac{1}{2}$, *the saddle connection breaks regularly with respect to the parameterAas one moves away from*$A_{\ell}(C)=-\frac{1}{16}+\frac{C}{4}$.

#### Proof

Recalling *V* from the proof of Proposition 2, we see that along $A_{\ell}=-\frac{1}{16}+\frac{C}{4}$, $\{V=0\}$ is invariant and hence contains the centre separatrix of $p_{a}$. It is easy to verify that when $C<\frac{1}{2}$, only the node $p_{-}$ lies on $\{V=0\}$, and when $C>\frac{1}{2}$ only the saddle $p_{+}$ lies there. In the second case, the node $p_{-}$ is found to lie in $\{V>0\}$. So, the unstable separatrix of the saddle in $\{V<0\}$ can only connect to $p_{n}$. The computation of the eigenvalues is direct.

Let us finally prove the regular breaking property, with a Melnikov-like approach that is adapted to planar dynamics; see [19]. Denoting $\dot{X}=f$, $\dot{Y}=g$, then

$$fg_{A} - gf_{A} = Y^{2} - X $$

which evaluates to $-\frac{Y}{2}-\frac{1}{8}+\frac{C}{2}$ along $\{V=0\}$. This function has a fixed sign on the separatrix from $Y=-\infty$ up to the saddle $p_{+}$ at $Y=C-\frac{1}{4}$. We can now directly refer to [19] (Proposition 5.7) where the regular breaking is related to a Melnikov computation, where the integrand is exactly $fg_{A}-gf_{A}$ (multiplied by a specific exponential that implies the convergence of the Melnikov integral). Since this function is sign-fixed, the related Melnikov integral is nonzero; see [23] for a generalisation of Melnikov theory to arbitrary dimensions. □

#### Theorem 4

*Let*$C_{\min}>\frac{1}{2}$. *There exists a parameter surface*$A_{\ell}(C,\varepsilon )=-\frac{1}{16}+\frac{C}{4} + O(\varepsilon )$, $C > C_{\min}$*along which a large*-*amplitude saddle homoclinic* ($\mathit{HOM}_{\ell}$) *connection exists*. *On this surface*, *there exists a curve*$C=\frac{3}{4} + O(\varepsilon )$*along which the homoclinic changes stability* (*resonant*$\mathit{HOM}_{\ell}$): *for lower values ofC*, *the homoclinic is stable*, *for larger values it is unstable*. *From this curve emerges a surface*$A=A_{\mathrm{snpo}}(C,\varepsilon )$*along which a SNPO bifurcation takes place*. *The surfaces*$A_{\mathrm {snpo}}(C,\varepsilon )$*and*$A_{\ell}(C,\varepsilon )$*are exponentially close*.

#### Proof

The presence of the homoclinic surface follows from a reasoning completely analogous to the one in the proof of Theorem 3. The change of stability is simply an eigenvalue computation: the equation $\rho(C)=-1$ is perturbed regularly under the *ε*-perturbation.

The emergence of an SNPO branch from the resonant saddle homoclinic is standard (see [24]), and based on three features: (i) the ratio of eigenvalues is perturbed regularly upon variation of a parameter (*C*), (ii) the separatrix connection breaks regularly upon variation of another parameter (*A*), and (iii) the divergence integral along the homoclinic loop is nonzero. Properties (i) and (ii) follow directly from the singular limit analysis in Proposition 3. Property (iii) follows from the slow–fast nature of the global return mechanism: the divergence integral computation is dominated by the passages along the slow branches $S_{a}^{\pm}$, which are both attracting and yield a contribution of the order $-K/\varepsilon $, for some $K>0$, while the fast parts and the parts near the folds yield an $O(1)$ contribution. While this argument does not prove that the SNPO branch is uniformly defined up to the limit, the proof of such a result is based on combining the local *Dulac* map of the saddle with a return mechanism. Since all properties are uniform and since the global return mechanism is sufficiently smooth up to and including the singular limit, the method for showing SNPO branches is valid uniformly in *ε*. □

Figure 10 summarises all observed codimension-2 bifurcations and the bifurcating codimension-1 branches.

#### Remark 15

There are no additional bifurcations (proof omitted).

#### Remark 16

The homoclinic surface $A_{\ell}(C,\varepsilon )$ defined in Theorem 4 can be extended to $C=\frac{1}{2}$, up to and including its intersection with the SN-surface $A_{\mathrm{sn}}^{+}(C,\varepsilon )$ from Theorem 3. At the singular limit, this is seen in Fig. 10, but a proof is needed for $\varepsilon >0$. In such a proof, one would need to blow up the vector field once more at the saddle-node singularity $(x,y) = (\frac{1}{16},\frac{1}{4})$ and at the parameter value $(a,c)=(\frac{1}{16},\frac{1}{2})$, using a family blow-up, in order to uniformly separate the saddle from the node. The technical issues involved in such a construction go beyond the scope of what we intend to expose in this paper.

#### Remark 17

The bifurcation curves AH, SNPO, $\mathrm{HOM}_{\ell}$ and $\mathrm{HOM}_{s}$ shown in Fig. 8 and Fig. 10 are the same. To rigorously prove this, we would need to include the parameters $(a,c)$ in the blow-up analysis, i.e. we would have to blow up the origin $(x,y,\varepsilon ,a,c)=(0,0,0,0,0)$. Again, the technicalities involved in such a construction go beyond the scope of what we intend to expose in this paper.

## Discussion

Excitability is an important subject area in neuroscience and its modern treatment dates back to Alan Hodgkin’s seminal work [1]. His distinction of three neural excitability classes based on injected steps of currents and observed corresponding distinct frequency–current (f–I) curves still forms the basis in understanding bifurcation mechanisms of neural excitability. FitzHugh was the first to use dynamical systems techniques for the qualitative description of action potential generation and threshold phenomena [7, 8, 25]. Rinzel and Ermentrout [3, 4] provided then a mathematical framework based on bifurcation theory to distinguish between these excitability types: SNIC bifurcation for type I and Andronov–Hopf bifurcation for type II.

This dynamical systems approach pioneered by Rinzel and Ermentrout is also used to explain more complicated neural activity such as bursting patterns. Here, the inherent multiple time-scale structure of neural models given through inherent slow and fast cell membrane processes is actively used to explain the bursting pattern. The bifurcation structure found in the fast subsystem provides a possible key to understanding the genesis of bursting patterns; see, e.g., [5]. Interestingly enough, the spiking pattern itself within a burst is also often a result of a multiple time-scale structure. This relaxation type behaviour is typically ignored in the bursting literature, because it would mean to consider models with (at least) three time scales—fast, intermediate and slow—which has become only recently a new research focus [26–28].

On the other hand, the literature on neural excitability (see, e.g. [5]) clearly uses slow–fast decomposition to explain action potential generation for tonic spiking models, although the accompanying bifurcation analysis of such a system often ignores or just inconsequently uses the given slow/fast structure. This article actively explores the singular nature of (two-dimensional) neural models and identifies a *novel* singular bifurcation based on the slow–fast structure—the *singular Bogdanov–Takens/SNIC* bifurcation—that is key to understanding type I and (part of) type II excitability. Using readily available tools and results from geometric singular perturbation theory [14–16, 19–22] and bifurcation theory [17, 24] we are able to unfold this singular bifurcation and identify important codimension-2 bifurcation points—Bautin, Bogdanov–Takens, resonant homoclinic and saddle-node homoclinic—which organise the bifurcation landscape for $\varepsilon >0$ and help to explain the transitions between type I and type II excitability. For example, based on the position of the Bautin point in parameter space we identify a supercritical Andronov–Hopf bifurcation as a clear indicator of type II excitability while a subcritical Andronov–Hopf bifurcation does not necessarily guarantee a frequency band (significantly) bounded away from zero, i.e. the model could be close to type I and thus close to a homoclinic. Another important indicator for this proximity to type I is the cusp bifurcation and its corresponding saddle-node branches. Within the cusp region there are three equilibria including a saddle which is necessary to form a homoclinic loop. In this type I–II transition regime, properties of model neurons that are considered type II might show behaviour usually associated with type I and vice versa. Care has to be taken when inferring properties from such a simple excitability classification; see also [5], where many of these bifurcations and observations have been highlighted.

Our analytical bifurcation results provide important information for the computational neuroscience community. We show that a SNIC bifurcation associated with type I excitability only exists in a small parameter regime. Thus it is more likely to observe a (large) saddle homoclinic in one parameter continuation of neural models, although it might be very close to a saddle-node and, hence, be mistaken for a SNIC. Similarly, if one observes a subcritical Andronov–Hopf bifurcation close to a saddle-node, especially where the corresponding periodic orbits terminate nearby in a (small) homoclinic, then the other observed (large) homoclinic that terminates close to a saddle-node cannot be a SNIC.

A main obstacle in a numerical bifurcation analysis is not only the stiffness of the underlying problem but also the close proximity of different bifurcation branches. Our analytical bifurcation results should be seen as a helpful guide for numerical continuation. For example, the numerical bifurcation diagrams presented, e.g., for the Morris–Lecar neural model in [29], Fig. 6, or for a 2D sodium spiking model in [30], Figs. 1–2, are incomplete since the exponentially close branches $\mathrm{HOM}_{s}$, $\mathrm{HOM}_{\ell}$, SNPO and SNIC (Fig. 8) are hard to distinguish numerically and the identification of certain codimension-2 points (Fig. 10)—Bogdanov–Takens, resonant $\mathrm{HOM}_{\ell}$ and $\mathrm{SN}\mbox{-}\mathrm{HOM}_{\ell}$—is a very difficult task.

## Footnotes

1 This is a condition on the position of possible equilibrium states in this model.

2 For example, it allows Assumption 1 (page 5) to be formulated independently of *λ*.

3 This first condition in (12) is in fact already a consequence from condition (10) together with (8).

4 With a slight abuse of notation we denote the new time derivative also by a prime.

5 This remark applies for all manipulations of the system in this proof.

6 In the canonical model (1), the *v*-coordinate of the fold does not depend on *I*.

7 Note that the cusp is not a ‘singular’ bifurcation since it persists in the singular limit as a bifurcation of the reduced problem, i.e. the cusp forms a regular bifurcation structure within this singularly perturbed system.

8 The cusp forms a regular bifurcation structure within this singularly perturbed system.

9 We refer to it as a singular Bogdanov–Takens point.

10 We refrain from writing the subindex 2 on $(X,Y)$ in this chart to distinguish the coordinates from $K_{1}$.

11 The separatrix of $p_{a}$ has an expansion $Y = -\sqrt{X}-\frac{1}{4} + \frac{1-8C}{32}X^{-1/2}- \frac {(2C-1)^{2}}{64}X^{-1} + O(X^{-3/2})$ as $X\to\infty$ and $V = -\frac {(2C-1)^{2}}{32}X^{-1/2} + O(X^{-1})$ along this separatrix.
